# 2,7-Dibromo-9,9-dimethyl-9*H*-fluorene

**DOI:** 10.1107/S1600536810012171

**Published:** 2010-04-10

**Authors:** Xiao Chen, Xinliang Fu, Yongkuan Qiu, Jialong Yuan

**Affiliations:** a Tianjin Basechem Technology Co. Ltd, K1-4-404, No. 6 Haitaifazhan 6th Road Huayuan Industry Area, Tianjin New Technology Industry Park, Tianjin 300384, People’s Republic of China

## Abstract

The title mol­ecule, C_15_H_15_Br_2_, has crystallographic *m*2*m* site symmetry. As a result, all atoms, except for those of the methyl groups, are exactly coplanar. In the crystal structure, there are weak π–π inter­actions with a centroid–centroid distance of 3.8409 (15) Å between symmetry-related mol­ecules, which stack along the *c* axis.

## Related literature

For applications of fluorene derivatives, see: Holder *et al.* (2005 ▶); Kulkarni *et al.* (2004 ▶); Padmaperuma *et al.* (2006 ▶); Seneclauze *et al.* (2007 ▶); Tsuboyama *et al.* (2003 ▶). For the properties of fluorene-based mol­ecules, see: Scherf & List (2002 ▶). For the synthesis of the title compound, see: Belfield *et al.* (2000 ▶).
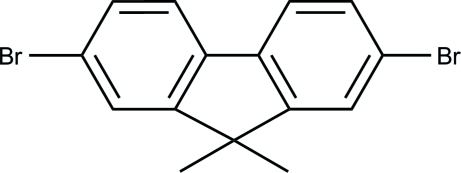


## Experimental

### 

#### Crystal data

C_15_H_12_Br_2_
*M* *_r_* = 352.07
Orthorhombic, 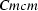

*a* = 17.097 (4) Å
*b* = 11.161 (3) Å
*c* = 6.9120 (17) Å
*V* = 1319.0 (6) Å^3^
*Z* = 4
Mo *K*α radiation
μ = 6.12 mm^−1^
*T* = 296 K
0.38 × 0.36 × 0.32 mm

#### Data collection

Bruker SMART CCD diffractometer
Absorption correction: multi-scan (*SADABS*; Sheldrick, 1996 ▶) *T*_min_ = 0.083, *T*_max_ = 1.000
3295 measured reflections
662 independent reflections
499 reflections with *I* > 2σ(*I*)
*R*_int_ = 0.047

#### Refinement

*R*[*F* ^2^ > 2σ(*F* ^2^)] = 0.032
*wR*(*F* ^2^) = 0.085
*S* = 1.05
662 reflections
54 parameters
H-atom parameters constrained
Δρ_max_ = 0.42 e Å^−3^
Δρ_min_ = −0.38 e Å^−3^

### 

Data collection: *SMART-NT* (Bruker, 1998 ▶); cell refinement: *SAINT-NT* (Bruker, 1998 ▶); data reduction: *SAINT-NT*; program(s) used to solve structure: *SHELXS97* (Sheldrick, 2008 ▶); program(s) used to refine structure: *SHELXL97* (Sheldrick, 2008 ▶); molecular graphics: *DIAMOND* (Brandenburg & Berndt, 1999 ▶); software used to prepare material for publication: *SHELXL97*.

## Supplementary Material

Crystal structure: contains datablocks global, I. DOI: 10.1107/S1600536810012171/lh5021sup1.cif

Structure factors: contains datablocks I. DOI: 10.1107/S1600536810012171/lh5021Isup2.hkl

Additional supplementary materials:  crystallographic information; 3D view; checkCIF report

## Figures and Tables

**Fig. 1.**
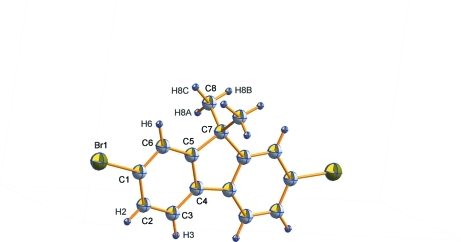
Molecular structure of title compound with the atom labeling of the asymmetric unit, showing displacement ellipsoids at the 30% probability level.

**Fig. 2.**
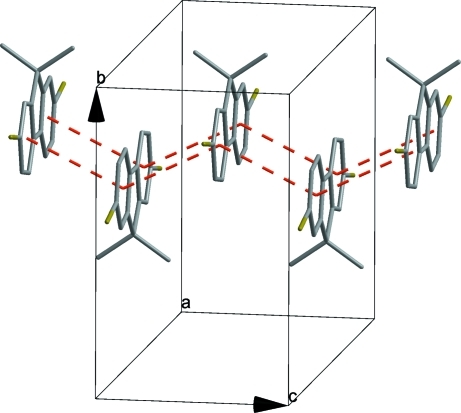
Part of the crystal structure with π–π interactions shown as red dashed lines.

## supplementary crystallographic information

### Comment

Because of their good thermal and chemical stability along with high emission efficiency, fluorene derivatives have shown many applications as electronic materials, especially for organic light emitting diodes (OLEDs) (Holder *et al.*, 2005; Kulkarni *et al.*, 2004; Seneclauze *et al.*, 2007; Padmaperuma *et al.*, 2006; Tsuboyama *et al.*, 2003). In this regard, small molecules, oligomers, or polymers with the 9,9-dialkylfluorene subunit possess interesting emissive properties. The quality and efficiency of such OLEDs have been shown to depend crucially on the stacking mode of the fluorene motif. On the other hand, the selected alkyl groups with different lengths or branched alkyl chains have a deep influence on the property and the packing mode of fluorene-based molecules (Scherf & List, 2002). During our study on such OLEDs crystalline materials, the crystal structure of the title compound has been determined in order to elucidate its molecular conformation and packing mode, which may be useful for further understanding its properties.

The molecular structure of the title compound is shown in Fig. 1. The complete molecule is generated two mirror planes which intersect each other [ crystallographic m2m site symmetry]. As a result, all the carbon atoms [except for those of the methyl groups] and the bromide atoms are exactly co-planar. In the crystal structure, weak π–π interactions between symmetry related benzene rings [C1-C6] with a centroid to centroid distance of 3.8409 (15) Å and perpendicular distance of 3.456 (1) Å form a one-dimensional chain along the *c* axis (see Fig. 2).

### Experimental

The title compound was prepared according to the literature method (Belfield *et al.*, 2000). Single crystals suitable for X-ray diffraction were obtained by recrystallization of a solution of the title compound in a mixture of ethyl acetate and petroleum ether.

### Refinement

H atoms were positioned geometrically and refined as riding atoms, with C—H = 0.96Å and U_iso_(H) = 1.5U_eq_(C) for methyl H atoms, and C—H = 0.93Å and U_iso_(H) = 1.2U_eq_(C) for all aromatic H atoms

### Crystal data

**Table:**

C_15_H_12_Br_2_	*F*(000) = 688
*M**_r_* = 352.07	*D*_x_ = 1.773 Mg m^−^^3^
Orthorhombic, *C**m**c**m*	Mo *K*α radiation, λ = 0.71073 Å
Hall symbol: -C 2c 2	Cell parameters from 958 reflections
*a* = 17.097 (4) Å	θ = 2.4–24.1°
*b* = 11.161 (3) Å	µ = 6.12 mm^−^^1^
*c* = 6.9120 (17) Å	*T* = 296 K
*V* = 1319.0 (6) Å^3^	Block, colourless
*Z* = 4	0.38 × 0.36 × 0.32 mm

### Data collection

**Table:**

Bruker SMART CCD diffractometer	662 independent reflections
Radiation source: fine-focus sealed tube	499 reflections with *I* > 2σ(*I*)
graphite	*R*_int_ = 0.047
φ and ω scans	θ_max_ = 25.0°, θ_min_ = 2.2°
Absorption correction: multi-scan (*SADABS*; Sheldrick, 1996)	*h* = −18→20
*T*_min_ = 0.083, *T*_max_ = 1.000	*k* = −13→11
3295 measured reflections	*l* = −7→8

### Refinement

**Table:**

Refinement on *F*^2^	Secondary atom site location: difference Fourier map
Least-squares matrix: full	Hydrogen site location: inferred from neighbouring sites
*R*[*F*^2^ > 2σ(*F*^2^)] = 0.032	H-atom parameters constrained
*wR*(*F*^2^) = 0.085	*w* = 1/[σ^2^(*F*_o_^2^) + (0.0321*P*)^2^ + 2.5078*P*] where *P* = (*F*_o_^2^ + 2*F*_c_^2^)/3
*S* = 1.05	(Δ/σ)_max_ < 0.001
662 reflections	Δρ_max_ = 0.42 e Å^−^^3^
54 parameters	Δρ_min_ = −0.38 e Å^−^^3^
0 restraints	Extinction correction: *SHELXL97* (Sheldrick, 2008), Fc^*^=kFc[1+0.001xFc^2^λ^3^/sin(2θ)]^-1/4^
Primary atom site location: structure-invariant direct methods	Extinction coefficient: 0.0097 (9)

### Special details

**Table:**

Geometry. All esds (except the esd in the dihedral angle between two l.s. planes) are estimated using the full covariance matrix. The cell esds are taken into account individually in the estimation of esds in distances, angles and torsion angles; correlations between esds in cell parameters are only used when they are defined by crystal symmetry. An approximate (isotropic) treatment of cell esds is used for estimating esds involving l.s. planes.
Refinement. Refinement of *F*^2^ against ALL reflections. The weighted *R*-factor wR and goodness of fit *S* are based on *F*^2^, conventional *R*-factors *R* are based on *F*, with *F* set to zero for negative *F*^2^. The threshold expression of *F*^2^ > σ(*F*^2^) is used only for calculating *R*-factors(gt) etc. and is not relevant to the choice of reflections for refinement. *R*-factors based on *F*^2^ are statistically about twice as large as those based on *F*, and *R*- factors based on ALL data will be even larger.

### Fractional atomic coordinates and isotropic or equivalent isotropic displacement parameters (Å^2^)

**Table:**

	*x*	*y*	*z*	*U*_iso_*/*U*_eq_	Occ. (<1)
Br1	0.19165 (3)	0.13056 (5)	0.2500	0.0696 (4)	
C1	0.3008 (3)	0.0990 (4)	0.2500	0.0416 (11)	
C2	0.3248 (3)	−0.0185 (4)	0.2500	0.0407 (12)	
H2	0.2883	−0.0804	0.2500	0.049*	
C3	0.4037 (3)	−0.0430 (4)	0.2500	0.0373 (11)	
H3	0.4210	−0.1220	0.2500	0.045*	
C4	0.4574 (3)	0.0500 (3)	0.2500	0.0318 (10)	
C5	0.4314 (3)	0.1684 (4)	0.2500	0.0332 (10)	
C6	0.3527 (3)	0.1946 (4)	0.2500	0.0392 (11)	
H6	0.3350	0.2734	0.2500	0.047*	
C7	0.5000	0.2560 (5)	0.2500	0.0373 (15)	
C8	0.5000	0.3344 (4)	0.0682 (8)	0.0533 (14)	
H8A	0.5000	0.2845	−0.0442	0.080*	
H8B	0.5481	0.3785	0.0613	0.080*	0.50
H8C	0.4569	0.3894	0.0736	0.080*	0.50

### Atomic displacement parameters (Å^2^)

**Table:**

	*U*^11^	*U*^22^	*U*^33^	*U*^12^	*U*^13^	*U*^23^
Br1	0.0319 (4)	0.0648 (5)	0.1121 (6)	0.0034 (3)	0.000	0.000
C1	0.026 (2)	0.048 (3)	0.051 (3)	0.001 (2)	0.000	0.000
C2	0.038 (3)	0.039 (3)	0.046 (3)	−0.006 (2)	0.000	0.000
C3	0.043 (3)	0.026 (2)	0.043 (3)	−0.0029 (19)	0.000	0.000
C4	0.034 (2)	0.029 (2)	0.033 (2)	0.0008 (18)	0.000	0.000
C5	0.036 (3)	0.028 (2)	0.036 (2)	−0.0029 (19)	0.000	0.000
C6	0.036 (3)	0.031 (2)	0.050 (3)	0.006 (2)	0.000	0.000
C7	0.032 (3)	0.027 (3)	0.053 (4)	0.000	0.000	0.000
C8	0.047 (3)	0.042 (2)	0.071 (4)	0.000	0.000	0.017 (3)

### Geometric parameters (Å, °)

**Table:**

Br1—C1	1.899 (4)	C5—C6	1.377 (6)
C1—C2	1.374 (7)	C5—C7	1.528 (6)
C1—C6	1.388 (7)	C6—H6	0.9300
C2—C3	1.377 (6)	C7—C5^i^	1.528 (6)
C2—H2	0.9300	C7—C8^ii^	1.531 (6)
C3—C4	1.386 (6)	C7—C8	1.531 (6)
C3—H3	0.9300	C8—H8A	0.9561
C4—C5	1.394 (6)	C8—H8B	0.9600
C4—C4^i^	1.457 (9)	C8—H8C	0.9600
			
C2—C1—C6	122.9 (4)	C5—C6—C1	117.5 (4)
C2—C1—Br1	118.1 (4)	C5—C6—H6	121.2
C6—C1—Br1	119.1 (4)	C1—C6—H6	121.2
C1—C2—C3	118.8 (4)	C5—C7—C5^i^	100.3 (5)
C1—C2—H2	120.6	C5—C7—C8^ii^	111.47 (14)
C3—C2—H2	120.6	C5^i^—C7—C8^ii^	111.47 (14)
C2—C3—C4	120.0 (4)	C5—C7—C8	111.47 (14)
C2—C3—H3	120.0	C5^i^—C7—C8	111.47 (14)
C4—C3—H3	120.0	C8^ii^—C7—C8	110.3 (5)
C3—C4—C5	119.9 (4)	C7—C8—H8A	109.5
C3—C4—C4^i^	131.5 (2)	C7—C8—H8B	109.5
C5—C4—C4^i^	108.6 (3)	H8A—C8—H8B	104.9
C6—C5—C4	120.9 (4)	C7—C8—H8C	109.5
C6—C5—C7	127.9 (4)	H8A—C8—H8C	113.8
C4—C5—C7	111.2 (4)	H8B—C8—H8C	109.5
			
C6—C1—C2—C3	0.0	C7—C5—C6—C1	180.0
Br1—C1—C2—C3	180.0	C2—C1—C6—C5	0.0
C1—C2—C3—C4	0.0	Br1—C1—C6—C5	180.0
C2—C3—C4—C5	0.0	C6—C5—C7—C5^i^	180.0
C2—C3—C4—C4^i^	180.0	C4—C5—C7—C5^i^	0.0
C3—C4—C5—C6	0.0	C6—C5—C7—C8^ii^	61.9 (3)
C4^i^—C4—C5—C6	180.0	C4—C5—C7—C8^ii^	−118.1 (3)
C3—C4—C5—C7	180.0	C6—C5—C7—C8	−61.9 (3)
C4^i^—C4—C5—C7	0.0	C4—C5—C7—C8	118.1 (3)
C4—C5—C6—C1	0.0		

Symmetry codes: (i) −*x*+1, *y*, −*z*+1/2; (ii) *x*, *y*, −*z*+1/2.
